# The Cysteine-Rich Repeat Protein TaCRR1 Participates in Defense against Both *Rhizoctonia* *cerealis* and *Bipolaris sorokiniana* in Wheat

**DOI:** 10.3390/ijms21165698

**Published:** 2020-08-09

**Authors:** Feilong Guo, Zilong Shan, Jinfeng Yu, Gangbiao Xu, Zengyan Zhang

**Affiliations:** 1 The Laboratory of Forestry Genetics, Central South University of Forestry and Technology, Changsha 410004, China; guofeilong1117@163.com; 2 The National Key Facility for Crop Gene Resources and Genetic Improvement, Institute of Crop Science, Chinese Academy of Agricultural Sciences, Beijing 100081, China; 3 ShiJiaZhuang Academy of Agricultural and Forestry Sciences, Shijiazhuang 050041, China; shanzil@163.com; 4 College of Plant Protection, Shandong Agricultural University, Taian 271018, China; jfyu@sdau.edu.cn

**Keywords:** antifungal activity, *Bipolaris sorokiniana*, cysteine-rich repeat protein, DUF26 domain, *Rhizoctonia cerealis*, wheat (*Triticumaestivum*)

## Abstract

The domain of unknown function 26 (DUF26), harboring a conserved cysteine-rich motif (C-X8-C-X2-C), is unique to land plants. Several cysteine-rich repeat proteins (CRRs), belonging to DUF26-containing proteins, have been implicated in the defense against fungal pathogens in ginkgo, cotton, and maize. However, little is known about the functional roles of CRRs in the important staple crop wheat (*Triticum aestivum*). In this study, we identified a wheat CRR-encoding gene *TaCRR1* through transcriptomic analysis, and dissected the defense role of *TaCRR1* against the soil-borne fungi *Rhizoctonia cerealis* and *Bipolaris sorokiniana*, causal pathogens of destructive wheat diseases. *TaCRR1* transcription was up-regulated in wheat towards *B. Sorokiniana* or *R. cerealis* infection. The deduced TaCRR1 protein contained a signal peptide and two DUF26 domains. Heterologously-expressed TaCRR1 protein markedly inhibited the mycelia growth of *B. sorokiniana* and *R. cerealis*. Furthermore, the silencing of *TaCRR1* both impaired host resistance to *B. sorokiniana* and *R*. *cerealis* and repressed the expression of several pathogenesis-related genes in wheat. These results suggest that the TaCRR1 positively participated in wheat defense against both *B. sorokiniana* and *R. cerealis* through its antifungal activity and modulating expression of pathogenesis-related genes. Thus, *TaCRR1* is a candidate gene for improving wheat resistance to *B. sorokiniana* and *R. cerealis*.

## 1. Introduction

Common wheat (*Triticum aestivum*) is one of the most widely cultivated and important staple crops in the world [1]. Thus, its high yield is necessary for global food security. The production of wheat is often seriously impacted by a wide variety of pathogens. *Bipolaris sorokiniana* (teleomorph *Cochliobolus* *sativus*), a soil-borne fungal pathogen, is the causal agent of common root rot [2], a serious root disease of wheat. *B. sorokiniana* can also cause leaf spot blotch, seedling blight, head blight, and black point of grains in wheat and barley and other species of cereals and grasses [3,4]. Additionally, sharp eyespot of wheat, mainly caused by the necrotrophic fungus *Rhizoctonia cerealis*, is one of the destructive diseases of wheat in some regions of the world [5]. Since 2005, wheat production on more than 6.67 million hectares each year has been harmed by sharp eyespot in China, where it has become one of the most serious wheat diseases in the world [5,6]. The effective and environmentally-friendly approach to control common root rot and sharp eyespot is to breed disease-resistant varieties. However, the disease resistance breeding is difficult by conventional methods because no completely resistant wheat accessions and few typical resistance genes are available [7]. Thus, it is necessary to identify effective resistance genes at a molecular level for breeding disease-resistance wheat cultivars with resistance to common root rot and sharp eyespot.

To combat numerous biotic stresses, plants have evolved two-layers of immune systems including pathogen-associated molecular pattern (PAMP)-triggered immunity (PTI) and effector-triggered immunity (ETI) [8]. Both responses lead to the production of defense molecules and pathogenesis-related (PR) proteins [8,9]. Among the defense molecules, plant chitinases can inhibit the growth of many fungi through hydrolysis of chitin, a major structural component, of the fungal cell wall. Chitinases also have the secondary effect of releasing chitin, which functions as a PAMP [9]. β-1,3-Glucanase is able to hydrolyze β-1,3-glucan linker, a major structural cell wall component of the fungal cell wall, and to suppress fungal growth; thus, the enzyme acts as a defense molecule against fungal pathogens in plants [10]. Defensin can permeabilize fungal plasma membranes, induce Ca^2+^ influx, and disrupt a Ca^2+^ gradient essential for polar growth of hyphal tips [11]. Some defensins bind with high affinity to specific sphingolipids present in the fungal cell wall or plasma membrane of their target fungi [12,13,14]. Thus, many defensins exhibit potent antifungal activity in vitro, inhibiting the growth of fungi and oomycetes at micromolar concentrations [11,12,13,14].

The domain of unknown function 26 (DUF26) harbors C-X8-C-X2-C (CRR) motif and is unique to plant systems. These conserved cysteine residues are predicted to form disulfide bonds, which are known to mediate protein–protein interactions [15] and to be targeted for apoplastic redox modification [16,17]. DUF26 was initially found in the extracellular regions of cysteine-rich receptor-like kinases (CRKs), plasmodesmata-located proteins (PDLP), and cysteine-rich repeat proteins (CRRs) [17,18]. In genomes of *Arabidopsis* and rice (*Oryza sativa*), there are ~100 and over 250 DUF26-containing protein-encoded genes, respectively [18]. The overexpression of several *Arabidopsis* CRKs, AtCRK4, AtCRK6, AtCRK13, AtCRK28 and AtCRK36 increases resistance to the pathogen *Pseudomonas syringae* [19,20]. In *Arabidopsis*, PDLP1 and PDLP5 (plasmodesmata-located proteins) have been implicated in the coordination of cell-to-cell communication and defense against the pathogens *Hyaloperonospora arabidopsidis* and *P. syringae*, respectively [21,22]. The Gnk2 from the gymnosperm *G. biloba,* comprising a single DUF26 domain, belongs to the CRR subfamily and inhibits the growth of fungi—such as *Fusariumoxy sporum*, *Trichoderma reesei*, and *Candida albicans*—through binding mannose in the cell walls of these fungi [23,24]. The cotton CRR1 protein (GbCRR1), comprising two DUF26 domains, interacts and protects host chitinase 28 from cleavage by secretory serine protease 1 of the fungal pathogen *Verticillium dahlia*. In cotton, knockdown of *GbCRR1* compromised plant resistance to *V. dahliae*, and *GbCRR1* overexpression increased plant resistance to *V. dahlia*, *Botrytis cinerea*, and the oomycete *Phytophthor aparasitica* var *nicotiana*e [25]. In maize, two CRR proteins—ZmAFP1 and ZmAFP2—interact with the effector Rsp3 (repetitive secreted protein 3) of the fungal pathogen *Ustilago maydis*. In addition, the ZmAFP1 displays antifungal activity through binding to mannose in the fungal cell wall and affecting the integrity of the fungal cell wall [26]. In wheat, the biological functions of CRRs remain unknown.

In this study, to explore if DUF26-containing CRRs are involved in wheat resistance responses to the soil-borne pathogens—*R. cerealis* and *B. sorokiniana,* we identified a wheat CRR-encoding gene *TaCRR1* in wheat defense responses to *R. cerealis* and *B. sorokiniana,* and validated the defense function of *TaCRR1.* Interestingly, the heterologously-expressed TaCRR1 protein obviously restrained the mycelia growth of *R. cerealis* and *B. sorokiniana*. Further functional investigation indicated that the knocking-down of TaCRR1 by virus-induced gene-silencing (VIGS) not only impaired host resistance to *B. sorokiniana* and *R. cerealis* but also repressed expression of pathogenesis-related genes in wheat, including *β-1,3-Glucanase*, *Defensin*, *Chitinase1*, *Chitinase3*, and *Chitinase4*. These results extend our understanding of wheat resistance mechanisms against the sharp eyespot caused by *R. cerealis* and common root rot caused by *B. sorokiniana*.

## 2. Results

### 2.1. TaCRR1 Transcription in Wheat Is Responsive to R. Cerealis or B. Sorokiniana Infection

**Figure 1:**
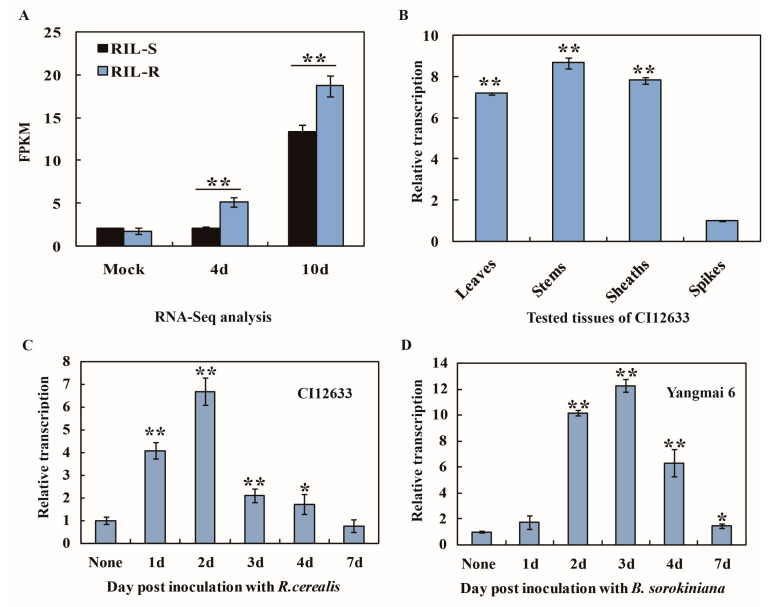
RNA-Seq and qRT-PCR analyses on transcriptional profiles of *TaCRR1* in the wheat responses to *Rhizoctonia cerealis* and *Bipolaris sorokiniana*. (**A**) RNA-Seq data of *TaCRR1* transcription in the resistant RILs (RIL-R) and in susceptible RILs (RIL-S) (use the same in text as in Figure 1A). (**B**) Transcription of *TaCRR1* in stems, leaves, sheaths, and spikes of CI12633 plants. The transcriptional level of *TaCRR1* in spikes was set to 1. (**C**) qRT-PCR analysis of *TaCRR1* in the resistant wheat Yangmai 6 plants inoculated with *B. sorokiniana*. (**D**) qRT-PCR analysis of *TaCRR1* in the resistant wheat cv. CI12633 plants inoculated with *R. cerealis*. The transcriptional level of *TaCRR1* at none (non-treatment) is set to 1. Statistically significant differences were derived from the results of three independent replications (*t*-test: * 0.01 < *p* < 0.05, ** *p* < 0.01). *TaGAPDH* was used as an internal control.

By means of analysis of RNA-Seq data from three resistant and three susceptible recombinant inbred lines (RILs) (derived from the cross of resistant wheat cultivar Shanhongmai and susceptible wheat cultivar Wenmai 6), we identified a wheat CRR gene sequence with ID TraesCS4A02G099000.1. Its transcriptional level was not only upregulated by infection of *R. cerealis* but it also was significantly higher in the resistant RILs (RIL-R) than in the susceptible RILs (RIL-S) at the same inoculating time points including mock (without the fungal inoculation) and 4 and 10 days post inoculated (dpi) with *R. cerealis* WK207 (Figure 1A). This gene was designated as *TaCRR1*. Further qRT-PCR results showed that the transcriptional level of *TaCRR1* was the highest in stems (sharp eyespot and common root rot mainly occurring tissue) and the lowest in spikes in the *R. cerealis*-resistant wheat cultivar (cv.) CI12633 plants (Figure 1B). At 2 dpi with *R. cerealis*, *TaCRR1* transcription was higher in the *R. cerealis*-resistant wheat cvs (CI12633 and Shanhongmai) than in the *R. cerealis*-susceptible wheat cvs (Yangmai 9 and Wenmai 6), and the highest expression level was detected in CI12633 (Figure S1). *TaCRR1* transcription in *R. cerealis*-resistant wheat cv. CI12633 was elevated toward *R. cerealis* infection, which reached a peak at 2 dpi (~6.67-fold) (Figure 1C). Interestingly, after infection of *B. sorokiniana,* the transcription of *TaCRR1* in *B. sorokiniana*-resistant wheat cv. Yangmai 6 was significantly increased at 2 dpi and reached a peak at 3 dpi (~12.25-fold) compared with untreated plants (Figure 1D). The expression profiles of *TaCRR1* indicated that the gene might be involved in wheat resistance responses to infection of *R. cerealis* and *B. sorokiniana*.

### 2.2. Sequence and Phylogenetic Analyses of TaCRR1

**Figure 2:**
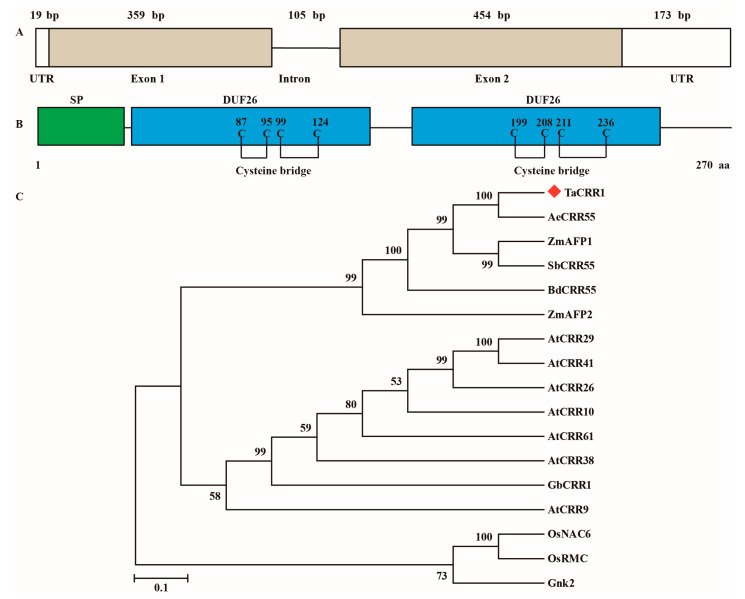
Sequence and phylogenetic analyses of TaCRR1. (**A**) Genomic structure of *TaCRR1* gene. White boxes indicate untranslated regions, grey boxes indicate exons, and the black line indicates an intron, respectively. (**B**) Schematic diagram of the domains (color areas) of TaCRR1 protein. (**C**) Phylogenetic analysis of TaCRR1 protein and 16 other cysteine-rich repeat (CRR) proteins. The bootstrapped phylogenetic tree is constructed using the neighbor-joining phylogeny of MEGA 7.0 (parameters: 1000 bootstraps).

**Figure 3:**
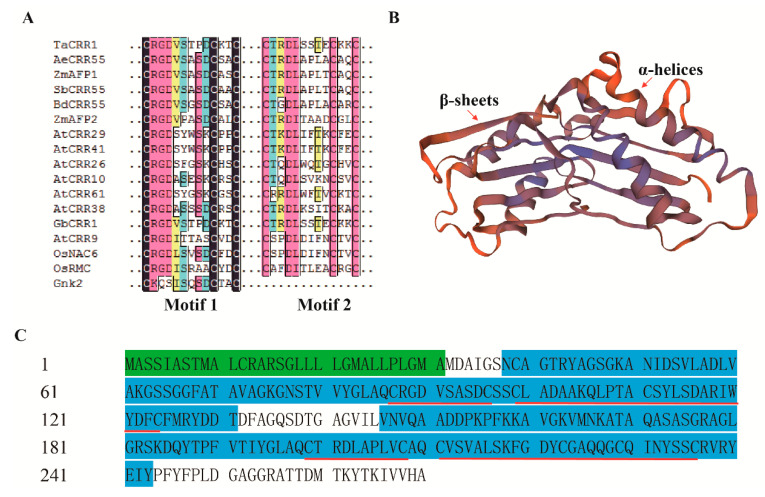
Domain and structure analyses of TaCRR1. (**A**) Amino acid alignment of CRR motifs between TaCRR1 and other CRR proteins. (**B**) The structure model of TaCRR1 protein. The structure of TaCRR1 protein was modeled using the Swiss-Model. (**C**) Schematic diagram of the domain (color area) and cysteine bridge of TaCRR1 protein. The green region indicated signal peptide domain; the blue region indicated DUF26 domain; the red underline indicated the cysteine bridge.

The full-length coding sequence and genomic sequence of *TaCRR1* were cloned from the *R. cerealis*-resistant wheat cv. CI12633 through two PCR rounds, respectively. *TaCRR1* contained an ORF (open read frame) with an 813-bp length (nucleotides 19 to 832 in the *TaCRR1* cDNA sequence) and was 100% matched to the wheat sequence with ID TraesCS4A02G099000.1. The comparison of the cDNA and genomic sequences showed that the genomic sequence of *TaCRR1* with 1110-bp length comprised an intron and two exons (Figure 2A). The deduced protein TaCRR1 consisted of 270 amino acid residues with a molecular weight of 28.13 kD and a theoretical isoelectric point (pI) of 8.51. As shown in Figure 2B and Figure 3, the TaCRR1 protein sequence contains a signal peptide domain (1–31 amino acids, aa) and two DUF26 domains (38–131 and 146–243 aa), which each harbor a C-X8-C-X2-C (cysteine-rich repeat, CRR) motif. Based on blast against the hexaploid wheat genome sequence (http://www.wheatgenome.org/, the Chinese Spring V2 genome), two additional homologues of *TaCRR1* were found on wheat chromosomes 4B and 4D from wheat cv. Chinese Spring (CS), named *TaCRR1-B* and *TaCRR1-D*, respectively. A pairwise comparison of these TaCRR1 protein sequences showed that TaCRR1 shared 93.33% and 95.93% identity with TaCRR1-B (262 aa) and TaCRR1-D (270 aa), respectively (Figure S2). The three copies of TaCRR1 all contained two DUF26 domains.

To investigate the phylogenetic relationship of TaCRR1 with other CRRs, we constructed a phylogenetic tree including TaCRR1, and 16 other CRR proteins, from *Arabidopsis*, rice, maize (*Zea mays*), *Aegilops tauschii*, *Brachypodium distachyum, Sorghum bicolor, Ginkgo biloba,* and cotton (*Gossypium barbadense*). The phylogenetic analysis showed that TaCRR1, AeCRR55 from *Ae. tauschii*, BdCRR55 from *B. distachyum*, SbCRR55 from *S. bicolor*, known immunity-related ZmAFP1, and known immunity-related ZmAFP2 from maize were clustered into the same clade. AtCRR9, AtCRR10, AtCRR26, AtCRR29, AtCRR38, AtCRR41, AtCRR61 from *Arabidopsis,* and GbCRR1 from cotton were clustered into the 2nd clade, while the third clade included Gnk2 from *G. biloba*, rice OsRMC, and OsNAC6 (Figure 2C). TaCRR1 had the highest sequence identity (82.59%) with an unknown function of AeCRR55. TaCRR1 shared 60.49% identity with ZmAFP1, a DUF26 protein with antifungal activity, and 33.55% identity with ZmAFP2. TaCRR1 shared 13.14%, 25.81%, 23.51%, and 20.30% identity with other known immunity-related CRR peptides, including Gnk2 in *G. biloba*, GbCRR1 in cotton, OsRMC and OsNAC6 in rice, respectively.

Bioinformatic analysis revealed that, except Gnk2 in *G. biloba*, all proteins of the above CRRs in *Arabidopsis*, rice, maize, wheat, *Ae. tauschii, B. distachyum*, *S. bicolor*, and cotton contained two copies of extracellular DUF26 domain with one CRR motif (Figure 3A). Based on the Swiss-Model, the modeled structure of TaCRR1 appears to be composed of two copies of DUF26 domain, each of which consists of two α- helices and five β-sheets (Figure 3B). In each DUF26 domain of TaCRR1, the four cysteine residues are predicted to form two cysteine bridges (Figure 2B, Figure 3C). The above data suggested that the TaCRR1 may be a novel cysteine-rich repeat protein in wheat and may be involved in plant innate immunity.

### 2.3. Heterologously Expressed TaCRR1 Inhibits the Mycelia Growth of B. sorokiniana and R. cerealis

**Figure 4:**
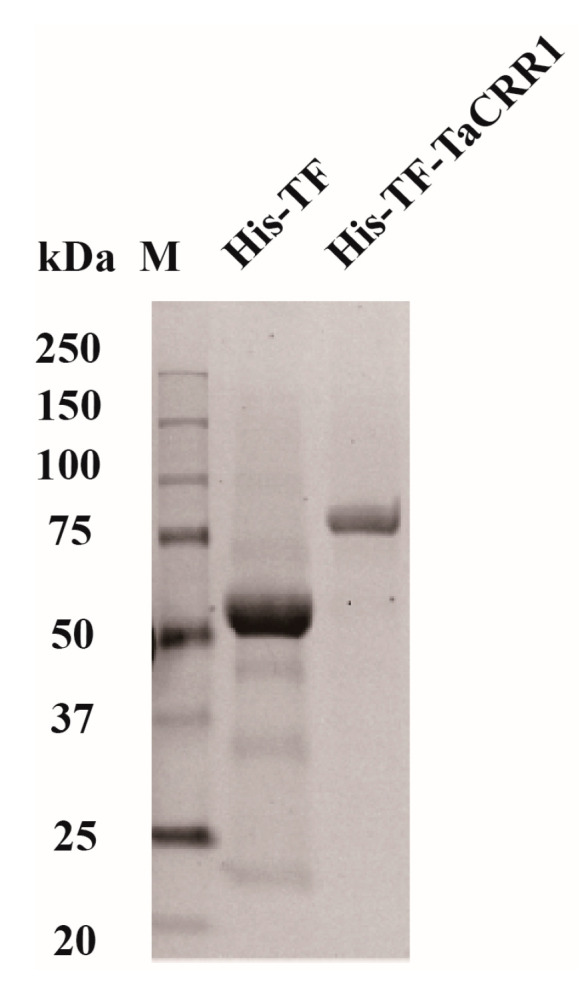
Heterologously-expressed TaCRR1 in *Escherichia coli*. Sodium dodecyl sulphate- polyacrylamide gel electrophoresis (SDS-PAGE) patterns of the purified His-TF and His-TF-TaCRR1 fusion proteins. His-TF is the control sample.

Elevated expression during the early infection stages indicates the important defense role of TaCRR1 against the fungi *R. cerealis* and *B. sorokiniana* in wheat. The TaCRR1 mature peptide was similar to that of ZmAFP1. In order to examine if the TaCRR1 protein inhibits the above pathogenic fungi, we constructed a recombinant protein expressing vector His-TF-TaCRR1, where the full sequence of TaCRR1 was subcloned in fusion to the 3′ terminal of the His-TF tag of pCOLD vector (Takara Bio Inc., Otsu, Japan). Subsequently, the His-TF-TaCRR1 recombinant protein and His-TF (control) peptide were transformed into *Escherichia coli* (DE3) cells, and highly expressed after culture and isopropyl-β-D-thiogalactopyranose (IPTG) induction. After purification, these His-TF-TaCRR1 and His-TF proteins were checked using sodium dodecyl sulphate-polyacrylamide gel electrophoresis (SDS-PAGE), and the purified recombinant protein His-TF-TaCRR1 migrated as a single band with estimated molecular masses of 80 kDa (Figure 4).

**Figure 5:**
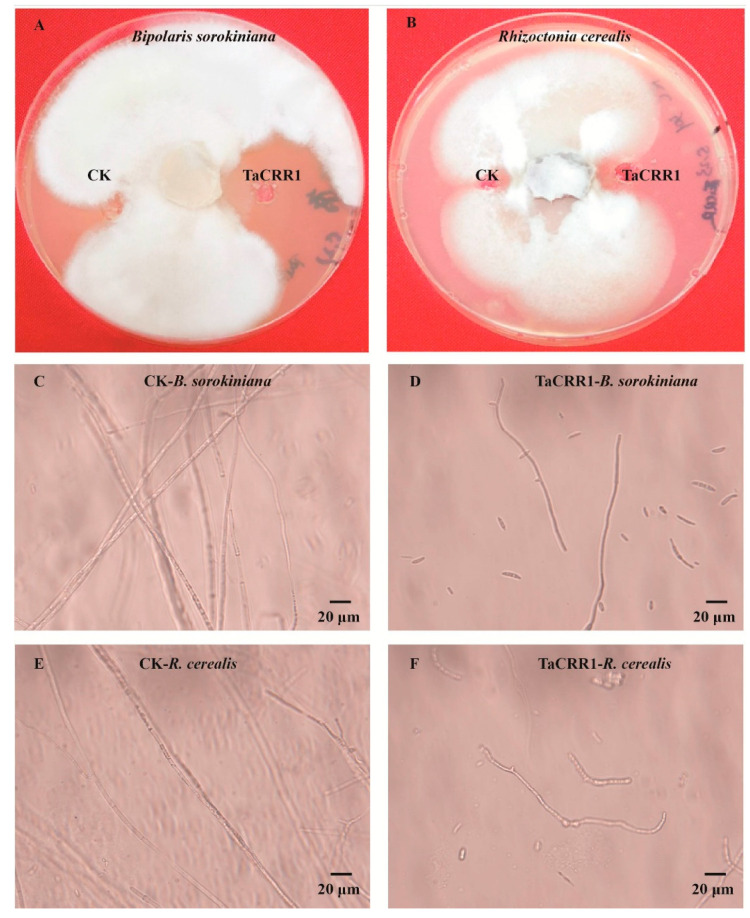
Antifungal activity assay of TaCRR1 against *Bipolaris sorokiniana* and *Rhizoctonia cerealis*. (**A**) Inhibiting effect of TaCRR1 protein on the mycelia growth of *B. sorokiniana*. The fungal mycelia were treated by 1 µmol L^−1^ His-TF (CK) and His-TF-TaCRR1 for 5 days. (**B**) Inhibiting effect of the TaCRR1 protein on the growth of *R. cerealis* mycelia. The fungal mycelia were treated by 1 µmol L^−1^ His-TF (CK) and His-TF-TaCRR1 for 5 days. (**C**) Photo by light microscopy (100×) of the hyphae of *B. Sorokiniana* treated by His-TF (CK) for 5 days. (**D**) Photo by light microscopy (100×) of the hyphae of *B. sorokiniana* treated by TaCRR1 for 5 days. (**E**) Photo by light microscopy (100×) of the hyphae of *R. cereals* treated by His-TF (CK) for 5 days. (**F**) Photo by light microscopy (100×) of the hyphae of *R. cerealis* treated by TaCRR1 for 5 days. The experiments were conducted three times. Scale bar = 20 μm.

When *B. sorokiniana* and *R. cerealis* mycelia plugs were separately inoculated in the center of the PDA medium and cultured for 2 days at 25 °C, the purified His-TF-TaCRR1 protein (1 μmolL^−1^) and control His-TF protein (CK, 1 μmolL^−1^) were separately injected into each small pore of the PDA medium. As a result, His-TF-TaCRR1 obviously inhibited the mycelia growth of *B. sorokiniana* and *R. cerealis* in these PDA media compared with the His-TF treatment (Figure 5A,B). Microscopy/photography images showed that the hyphae of *B. sorokiniana* on the edge treated by His-TF control (CK) were intact and well developed (Figure 5C), whereas the hyphae of *B. sorokiniana* on the edges of the inhibition zones treated with His-TF-TaCRR1 were deformed and lysed (Figure 5D). In addition, the antifungal activity to *R. cerealis* could be observed by microscope (Figure 5E,F). These results suggested that TaCRR1 protein restrained the mycelia growth of *B. sorokiniana* and *R. cerealis*.

### 2.4. Silencing of TaCRR1 Impairs Wheat Resistance to B. sorokiniana

**Figure 6:**
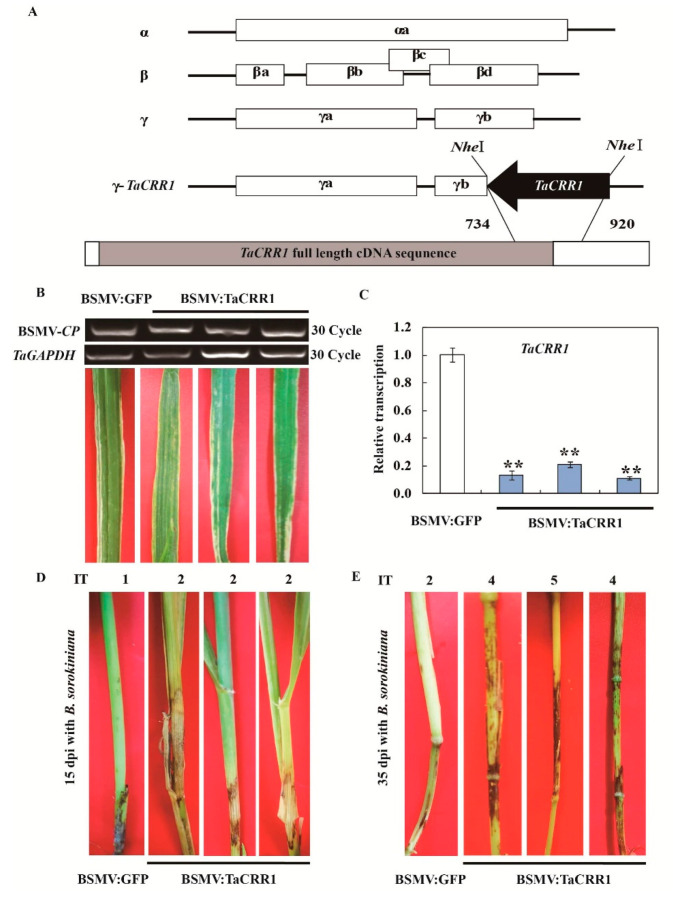
Silencing of *TaCRR1* by barley stripe mosaic virus (BSMV)-induced gene silencing impairs resistance of the resistant wheat cv. Yangmai 6 to *Bipolaris sorokiniana*. (**A**) Scheme of genomic RNAs of the barley stripe mosaic virus (BSMV) and the construct BSMV: TaCRR1 of the recombinant virus expressing the wheat gene *TaCRR1*. The orientation of the *TaCRR1* insert is indicated by dark boxes. (**B**) RT-PCR analysis on the transcription of BSMV coat protein (*CP*) in the wheat plants infected by BSMV: GFP or BSMV: TaCRR1 for 10 days. Mild chlorotic mosaic symptoms were observed on leaves of wheat Yangmai 6 plants at 10 dpi with BSMV: GFP or BSMV: TaCRR1. (**C**) qRT-PCR analysis of the *TaCRR1* gene in the wheat plants infected by BSMV: GFP or BSMV: TaCRR1 for 10 days. The relative transcript level of *TaCRR1* in BSMV: TaCRR1- infected wheat Yangmai 6 plants is relative to that in BSMV: GFP-infected Yangmai 6 (control) plants (set to 1). *TaGAPDH* was used as an internal control. Three replicates were averaged and statistically treated (*t*-test: **p < 0.01). Bars indicate the standard error of the mean. (**D**) Common root rot symptoms on sheaths and stems of the control and *TaCRR1*-silenced Yangmai 6 plants at 15 dpi with *B. sorokiniana*. (**E**) Common root rot symptoms on stems of the control and *TaCRR1*-silenced Yangmai 6 plants at 35 dpi with *B. sorokiniana*.

To investigate the defense function of *TaCRR1* by means of barley stripe mosaic virus (BSMV)-based VIGS, the VIGS fragment specific to *TaCRR1* was predicted by the Si-Fi software. Since the VIGS fragment of *TaCRR1* shared 100.00%, 83.67%, and 83.47% identity with those of *TaCRR1A, TaCRR1B,* and *TaCRR1D*, respectively, the Si-Fi prediction showed that *TaCRR1A* had more efficient siRNA hits compared with *TaCRR1B* and *TaCRR1D*. No off-target was predicted for the VIGS construct in wheat, as determined by the Si-Fi software. The VIGS fragment (186 bp) spanning the 3′ terminal of ORF and the 3′ untranslated region (UTR) of *TaCRR1* was sub-cloned in an antisense orientation into the *Nhe*I restriction site of RNA γ of BSMV and generated the BSMV-based VIGS vector (Figure 6A). At 10 days after the transfection of BSMV-derived RNAs into leaves of the *B. sorokiniana*-resistant wheat cv. Yangmai 6, symptoms of BSMV infection appeared on the newly emerged leaves, and the transcript of BSMV coat protein (*CP*) could be detected by RT-PCR (Figure 6B), indicating that BSMV indeed infected these wheat plants. The transcriptional level of *TaCRR1* was significantly decreased in BSMV: *TaCRR1-*infected Yangmai 6 plants compared to BSMV: GFP-infected Yangmai 6 plants (Figure 6C), suggesting that *TaCRR1* was successfully knocked-down in BSMV: TaCRR1-infected (*TaCRR1*-silenced) plants.

Further *TaCRR1*-silenced and BSMV: GFP-infected Yangmai 6 plants were inoculated with *B. sorokiniana*. At 15 dpi with the fungus, the sheaths of *TaCRR1*-silenced Yangmai 6 plants exhibited more serious necrosis of common root rot disease symptoms compared to BSMV:GFP-infected (control) plants (Figure 6D). At 35 dpi with *B. sorokiniana*, the stems of *TaCRR1*-silenced Yangmai 6 plants displayed more serious symptoms of common root rot disease than did BSMV: GFP-infected (control) plants (Figure 6E). Accordingly, the average infection type (IT) of TaCRR1-silenced Yangmai 6 plants was 3.64, and their common root rot disease index (DI) was 72.8, whereas the average IT and disease indexes of BSMV: GFP-treated Yangmai 6 plants were 1.82 and 36.4. These results indicated that the down-regulation of *TaCRR1* transcript in Yangmai 6 wheat impaired host resistance to *B. sorokiniana*, and TaCRR1 expression is required for the host resistance to common root rot caused by *B. sorokiniana* infection.

### 2.5. Silencing of TaCRR1 Reduces Wheat Resistance to R. cerealis

**Figure 7:**
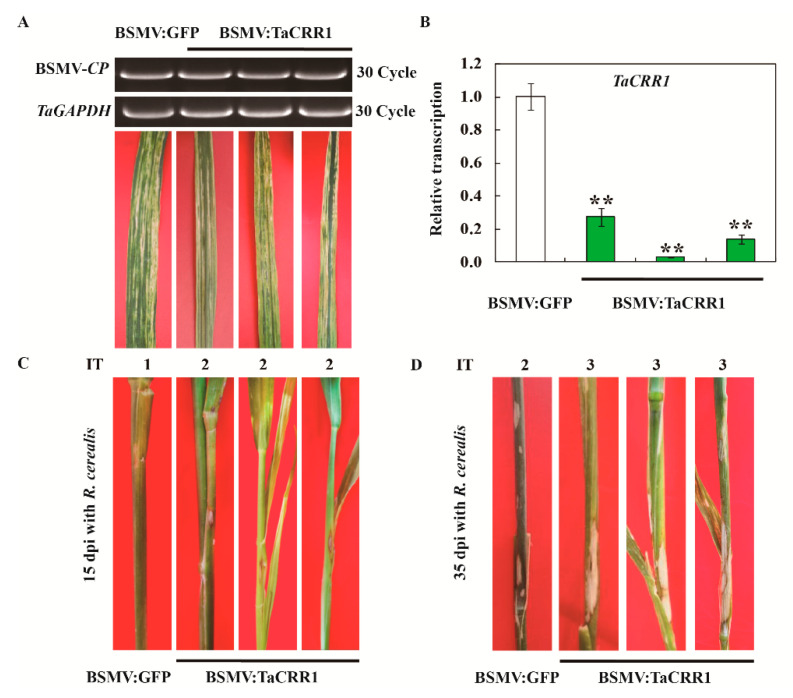
Silencing of *TaCRR1* by barley stripe mosaic virus (BSMV)-induced gene silencing impairs resistance of the resistant wheat cv. CI12633 to *Rhizoctonia cerealis*. (**A**) RT-PCR analysis of the transcription of BSMV coat protein (*CP*) in the wheat plants infected by BSMV: GFP or BSMV: TaCRR1 for 10 d. Mild chlorotic mosaic symptoms were observed on leaves of wheat CI12633 at 10 dpi with BSMV: GFP or BSMV: TaCRR1. (**B**) qRT-PCR analysis of the *TaCRR1* gene in the wheat plants infected by BSMV: GFP or BSMV: TaCRR1 for 10 days. The relative transcript level of *TaCRR1* in BSMV: TaCRR1-infected wheat CI12633 plants is relative to that in BSMV: GFP-infected CI12633 (control) plants (set to 1). *TaGAPDH* was used as an internal control. Three replicates were averaged and statistically treated (*t*-test: ** *p* < 0.01). Bars indicate the standard error of the mean. (**C**) Sharp eyespot symptoms on sheaths and stems of the control and *TaCRR1*-silenced CI12633 plants at 15 dpi with *R. cerealis*. (**D**) Sharp eyespot symptoms on stems of the control and *TaCRR1*-silenced CI12633 plants at 35 dpi with *R. cerealis*.

To further examine whether TaCRR1 expression was required for wheat resistance to *R. cerealis*, VIGS was deployed to knock-down the *TaCRR1* transcript in the *R. cerealis*-resistant wheat cv. CI12633. Following inoculation with BSMV: TaCRR1 or BSMV:GFP viruses for 10 days, the mild chlorotic mosaic symptoms of BSMV appeared in the leaves of the infected CI12633 plants, and the expression of the BSMV *CP* gene was detected (Figure 7A), proving that these inoculated CI12633 plants were successfully infected by BSMV. The qRT-PCR result showed that compared to BSMV: GFP infection, the transcript of *TaCRR1* was markedly knocked-down in BSMV: TaCRR1-infected CI12633 plants (Figure 7B). The *R*. *cerealis* strain WK207 was inoculated between the sheaths and the stems of these BSMV-infected plants to evaluate the role of *TaCRR1* in resistance to sharp eyespot. Following inoculation with *R*. *cerealis* for 10 and 35 days, sharp eyespot symptoms in *TaCRR1* silenced CI12633 plants were more serious than in control plants (Figure 7C,D). As expected, the average IT and sharp eyespot disease index of *TaCRR1*-silenced CI12633 plants were 2.36 and 47.2, respectively, whereas the average IT and disease index of BSMV: GFP-treated CI12633 plants were 1.61 and 32.2. The statistical analyses indicated that the silencing of *TaCRR1* in CI12633 impaired resistance of the host plants to sharp eyespot, suggesting that *TaCRR1* expression is required for defense of wheat at least the resistant wheat cv. CI12633 against sharp eyespot caused by *R. cerealis*.

### 2.6. Silencing of TaCRR1 Represses Expression of Several Pathogenesis-Related Genes

**Figure 8:**
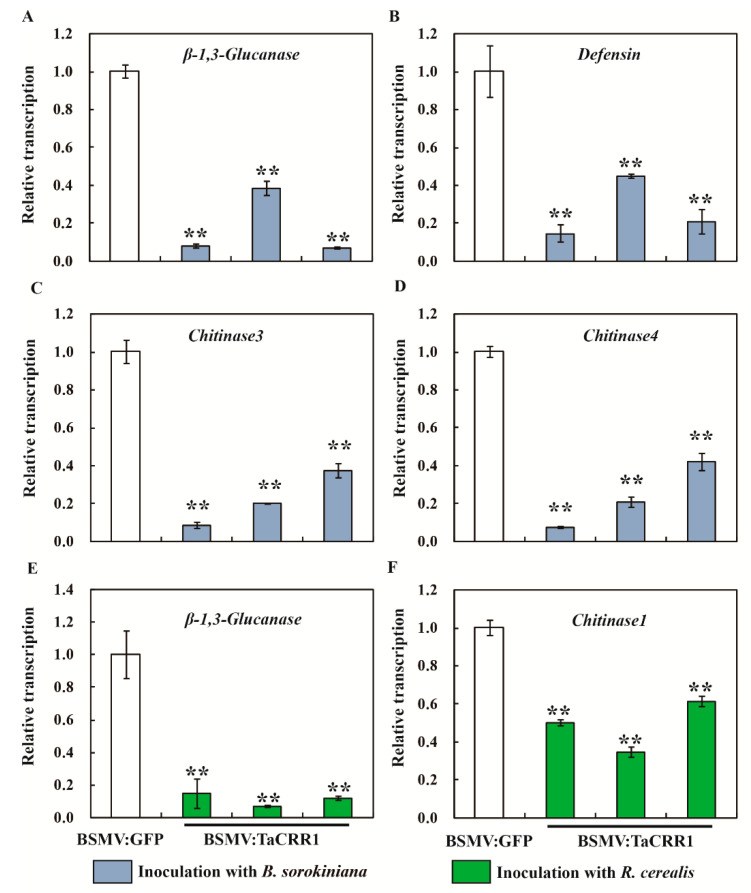
Transcription profiles of pathogenesis-related genes *β-1,3-Glucanase*, *Defensin*, *Chitinase1*, *Chitinase3,* and *Chitinase4* in BSMV: GFP- and BSMV: TaCRR1-infected wheat plants after infection by pathogens. Relative transcriptional abundances of the tested genes *β-1,3-Glucanase* (**A**), *Defensin* (**B**), *Chitinase3* (**C**), and *Chitinase4* (**D**) in BSMV: TaCRR1-infected Yangmai 6 plants were quantified relative to those in BSMV: GFP-infected control plants after *B. sorokiniana* inoculation for 15 days. Relative transcriptional abundances of the tested genes *β-1,3-Glucanase* (**E**) and *Chitinase1* (**F**) in BSMV: TaCRR1-infected CI12633 plants were quantified relative to that in BSMV: GFP-infected control plants after *R. cerealis* inoculation for 15 days. Statistically significant differences between BSMV: TaCRR1-infected and BSMV: GFP-infected wheat plants were determined based on three replications using a Student’s t-test (*t*-test: * 0.01 < *p* < 0.05; ** *p* < 0.01). Bars indicate the standard error of the mean. *TaGAPDH* was used as an internal control.

To investigate if *TaCRR1* is required for the expression of pathogenesis-related genes in wheat, qRT-PCR was used to examine the transcriptional levels of wheat pathogenesis-related genes in *TaCRR1-*silenced wheat and the BSMV: GFP-infected control plants inoculated 15 days previously with *B. sorokiniana* and *R*. *cerealis*, respectively. The tested genes included *β-1,3-Glucanase*, *Defensin*, *Chitinase1*, *Chitinase3*, and *Chitinase4*, which have been shown to participate positively in wheat resistance responses to *B. sorokiniana* and *R*. *cerealis* [27,28,29,30]. Fifteen days after inoculation with *B. sorokiniana*, the transcriptional levels of *β-1,3-Glucanase*, *Defensin*, *Chitinase3,* and *Chitinase4* (Figure 8A–D) were significantly decreased in *TaCRR1*-silenced Yangmai 6 plants relative to the BSMV: GFP-infected Yangmai 6 plants. Fifteen days after inoculation with *R*. *cerealis,* transcriptional levels of *β-1,3-Glucanase* and *Chitinase1* (Figure 8E–F) were significantly decreased in *TaCRR1*-silenced CI12633 plants relative to the BSMV: GFP-infected CI12633 plants. These data suggested that *TaCRR1* positively regulated the expression of these pathogenesis-related genes in wheat defense responses to *B. sorokiniana* and *R*. *cerealis*.

## 3. Discussion

Common root rot (*B. sorokiniana*) and sharp eyespot (*R. cerealis*) are economically important diseases in global wheat production. It is of great importance to identify new resistance genes from the wheat genome for improving wheat resistance to both diseases. In this study, the wheat cysteine-rich repeat protein gene *TaCRR1* was identified based on the RNA-seq-based transcriptomic data and qRT-PCR analysis. The transcription of *TaCRR1* in wheat was significantly elevated not only after *R. cerealis* infection but also by *B. sorokiniana* challenge. These results imply that *TaCRR1* might participate in wheat resistance responses to the common root rot and sharp eyespot pathogens. VIGS is a simple and powerful tool that can analyze gene function in a relatively rapid time frame [31,32,33]. For example, Scofield et al. deployed a BSMV-VIGS system to characterize the functional role of the leaf rust resistance gene *Lr21* in wheat [34]. BSMV-based silencing of *TaRCR1* (encoding a nucleotide-binding leucine-rich repeat protein) impaired resistance to *R. cerealis* in wheat [35]. By means of BSMV-VIGS, Zhang et al. analyzed the function of *TaNTF2* during an incompatible wheat-*Pucciniastrii* *formis*f. sp. *Tritici* (*Pst*). When *TaNTF2* was knocked down in wheat, the resistance of the host wheat plants to a virulent *Pst* decreased, with bigger necrotic spots, and higher numbers of hyphal branches and haustorial mother cells [36]. In the current report, we generated *TaCRR1-*silenced wheat plants by VIGS experiments and assessed the functional roles of *TaCRR1* in wheat defense against infection of *R. cerealis* and *B. sorokiniana*. The functional analysis results suggest that *TaCRR1* expression is required for wheat resistance responses to infection of the necrotrophic fungal pathogen *R. cerealis* and soil-born fungal pathogen *B. sorokiniana*. This study extends the current knowledge of plant CRRs in immune responses.

The heightened expression of pathogenesis-related genes, including Chitinases, β-1,3-Glucanases and defensins, positively contribute to plant defense against pathogens. Chitinases can inhibit growth of many pathogenic fungi through hydrolysis of chitin that is the major structural component of fungal cell walls [37]. β-1,3-Glucanases preferentially hydrolyze the β-1,3-glucan linker in the cell walls of many pathogenic fungi, including *Rhizoctonia solani*, *R. cerealis*, *Phytophthora capsici*, and *Alternaria longipes* [38]. Many plant defensins exhibit potent antifungal activity in vitro, inhibiting the growth of fungi and oomycetes at micromolar concentrations [11,12,13,14]. Previous studies indicated that several PR-encoding genes, such as *β-1,3-Glucanase*, *Defensin*, *Chitinase1*, *Chitinase3,* and *Chitinase4*, contributed to the resistance of wheat to *B. sorokiniana* and *R*. *cerealis* [27,28,29,30]. To explore the molecular mechanism underlying the defensive role of TaCRR1, we investigated the transcriptions of a subset of pathogenesis-related genes in *TaCRR1*-silenced wheat and the control plants. The results showed that after *B. Sorokiniana* inoculation, the transcriptional levels of *β-1,3-Glucanase*, *Defensin*, *Chitinase3,* and *Chitinase4* were down-regulated in more-susceptible *TaCRR1*-silenced Yangmai 6 plants than in the control plants. After *R. cerealis* inoculation, the transcriptional levels of *β-1,3-Glucanase* and *Chitinase1* were down-regulated in more-susceptible *TaCRR1*-silenced CI12633 plants than in the control wheat plants. The data imply *TaCRR1* modulated different defense molecules in the wheat resistance responses of CI12633 and Yangmai 6 against *R*. *cerealis* and *B. sorokiniana*.

In the current study, deduced TaCRR1 is a basic (pI 8.51) peptide and contains two extracellular DUF26 domains, belonging to the cysteine-rich repeat DUF26 proteins. Each of its DUF26 domains is an extracellular domain harboring a conserved CRR motif (C-8X-C-2X-C), which is a common feature among many antimicrobial proteins. For instance, the DUF26 peptide Gnk2 from *G. biloba* inhibits the growth of several fungi through binding mannose of these fungal cell walls [23,24]. In cotton, the GbCRR1 protein has been implicated in resistance against *Verticillium dahli* through protecting host chitinase 28 from cleavage by secretory Serine protease 1 of the fungal pathogen [25]. Our antifungal activity assay results indicated that the heterologously-expressed TaCRR1 protein obviously inhibited mycelia growth of the two soil-borne fungi *R. cerealis* and *B. sorokiniana*, demonstrating that TaCRR1 has a direct antifungal activity. In maize, ZmAFP1 interacts with the *Ustilago maydis* repetitive effector *Rsp3* and binds to mannose in the fungal cell wall, which affects the integrity of the fungal cell wall and consequently exhibits antifungal activity [26]. The current phylogenetic tree and sequence analyses indicated that TaCRR1 shares 60.49% identity with ZmAFP1, both of which possess two copies of DUF26 domain with C-8X-C-2X-Cmotif. Taken together, we suppose that TaCRR1 exhibits antifungal activity possibly through binding to mannose in these fungal cell walls.

In summary, we identified the novel cysteine-rich repeat protein gene *TaCRR1* in the wheat defense responses to *B. sorokiniana* and *R. cerealis* infection. The heterologously-expressed TaCRR1 protein can directly inhibit the mycelia growth of *B. sorokiniana* and *R. cerealis*. The *TaCRR1* expression is required for both wheat resistance responses to *B. sorokiniana* and *R. cerealis* and the expression of several pathogenesis-related genes, including *β-1,3-glucanase*, *Defensin*, *Chitinase1*, *Chitinase 3,* and *Chitinase 4*. Thus, the expression of TaCRR1 mediates immune responses to *B. sorokiniana* and *R. cerealis* through antifungal activity and modulating the expression of pathogenesis-related genes in wheat. To our knowledge, this is the first investigation into the functional role of CRRs in wheat. This study provides new insight into the roles of CRRs in plant innate immunity, especially immune responses of cereal crop plants to pathogens. *TaCRR1* is a promising gene that can be used to improve resistance of wheat to *B. sorokiniana* and *R. cerealis* infection.

## 4. Materials and Methods

### 4.1. Plant and Fungal Materials, and Primers

Four wheat (*Triticum aestivum*) cultivars—CI12633, Shanhongmai, Wenmai 6, and Yangmai 9—exhibiting different levels of resistance and susceptibility to sharp eyespot [6,7], were used to analyze the *TaCRR1* transcript profile. The *R. cerealis*-resistant wheat cv. CI12633 and *B. sorokiniana*-resistant wheat cv. Yangmai 6 were used in investigation of the defense function of *TaCRR1* against sharp eyespot and common root rot by means of VIGS experiment, respectively. Three resistant and three susceptible wheat lines of recombinant inbred lines derived from the cross Shanhongmai × Wenmai 6 and provided by Prof. Jizeng Jia (ICS, CAAS), were used for RNA-Seq and transcriptomic analyses.

The pathogenic fungi including *R. Cerealis* strains WK207 and R0301, and the *B. Sorokiniana* strain WB112, were isolated and provided by Profs. Jinfeng Yu (Shandong Agricultural University, China) and Huaigu Chen (Jiangsu Academy of Agricultural Sciences, China). These fungal strains were grown on potato dextrose agar (PDA) at 4 °C in the dark. To conduct disease tests/antifungal activity, subcultures were made on new PDA plates or potato dextrose liquid culture, which were then cultivated at 25 °C for 7–10 days before inoculation.

All plants of the wheat cultivars were grown in field plots or in the greenhouse under 14 h light (23 °C)/ 10 h dark (12 °C) conditions [6]. For the transcription pattern analysis of *TaCRR1* in wheat responses to *R. cerealis* and *B. sorokiniana* at the tillering stage, plants were inoculated between the base sheaths and the base stems of CI12633 and Yangmai 6 plants with toothpick fragments harboring well-developed mycelia of *R. cerealis* or *B. sorokiniana*, respectively. The inoculated sheaths and stems were sampled at nontreatment, 1, 2, 3, 4, and 7 dpi; rapidly frozen in liquid nitrogen; and stored at −80 °C.

The sequences of all primers in this study are listed in Table 1.

### 4.2. Cloning and Sequence Analysis of TaCRR1

For cloning DNA and cDNA sequences, specific primers of *TaCRR1* were designed based on the identified candidate sequence TraesCS4A02G099000.1. The DNA sequence of *TaCRR1* was amplified in two rounds of nested PCR from the DNA of sheaths/stems of *R. cerealis*-resistant wheat CI12633. Primers for the first round of PCR were TaCRR1-F1 and TaCRR1-R1, and those for the second round were TaCRR1-F2 and TaCRR1-R2. The cDNA sequence of TaCRR1 was amplified in two rounds of nested PCR from the cDNA of CI12633 sheaths/stems at the 2 dpi *R. cerealis* strain WK207. The PCR products were cloned into pMD18-T vector (Takara Bio Inc., Otsu, Japan) and then sequenced.

The cDNA sequence of *TaCRR1* was analyzed using the ORF finder (https://www.ncbi.nlm.nih.gov/orffinder/). Pfam database (http://pfam.xfam.org/) and smart software (http://smart.embl-heidelberg.de/) were used to analyze the deduced protein sequence of TaCRR1. The structure of the TaCRR1 protein modeling was performed with the Swiss-Model (https://swissmodel.expasy.org/interactive). Cysteine bridges were predicted by using http://prosite.expasy.org/. Sequence alignment was performed by DNAMAN software. A neighbor-joining phylogenetic tree was constructed using the MEGA V7.0 program with 1000 bootstrap replications.

### 4.3. RNA Extraction, cDNA Synthesis and (q)RT-PCR

To analyze the specific expression of *TaCRR1* from wheat plants of all cultivars, the Total RNA was extracted using Trizol reagent (Invitrogen, Life Technologies, Carlsbad, CA, USA) according to the manufacturer’s instructions. First-strand cDNA was synthesized using the FastQuant RT Kit (Tiangen, Beijing, China) for RT-PCR or qRT-PCR.

Specific primers of *TaCRR1*, *β-1,3-Glucanase*, *Defensin*, *Chitinase1*, *Chitinase3*, and *Chitinase4* of wheat and *CP* of BSMV were used to analyze, by (q)RT-PCR, the expression of these genes. qRT-PCR reactions were performed using a SYBR Premix Ex Taq kit (Takara Bio Inc., Otsu, Japan) in an ABI 7500 real time PCR system/instruction (Applied Biosystems, Waltham, MA, USA). qRT-PCR data were analyzed using the comparative 2^−ΔΔCT^ method [39], where the wheat *GAPDH* gene *TaGAPDH* was used as an internal reference. Each treatment included three independent replicates.

### 4.4. Heterologous Expression and Purification of TaCRR1

The full coding sequences of the *TaCRR1* gene were sub-cloned into the *BamH*I site of pCOLD vector (Takara Bio Inc., Otsu, Japan) and fused with the His-Trigger Factor (His-TF) tag in the vector, which resulted in the expression vectors His-TF-TaCRR1. The positive clones with the His-TF-TaCRR1 recombinant genes were identified by means of gene-specific PCR and confirmed by sequencing of the respective genes. The resulting His-TF-TaCRR1 fusion constructs were transformed into competent cells of *E. coli* BL21 (DE3). The recombinant His-TF-TaCRR1 and His-TF, were separately expressed after treatment with 0.5 mM IPTG at 16 °C for 19 h, and purified using Ni-NTA resin (TransGen Biotech, Beijing, China). Protein purity and molecular weight were determined by using SDS-PAGE according to the method described by Lu et al. [40].

### 4.5. Antifungal Activity Assay of TaCRR1 In Vitro

According to the mycelia growth inhibition method [38], the *R. cerealis* and *B. sorokiniana* mycelia plugs were separately inoculated in the center of one PDA plate and then cultured for 2 days at 25 ℃. Two symmetrical pores were punched in the medium, and His-TF-TaCRR1 and His-TF were separately injected into one pore, respectively, and further cultured at 25℃ for 5 days. The mycelia growth was observed. All treatments were repeated three times. *R. cerealis* and *B. sorokiniana* hyphae from the edges of the zones of inhibited mediums produced by the purified His-TF-TaCRR1, or His-TF, were scraped and observed under a light microscope (100×) for morphological changes.

### 4.6. BSMV-Mediated TaCRR1 Gene Silencing in Wheat Plants and Their Disease Assessment

The 186bp-length fragment specific of *TaCRR1* (no. 734 to 920 nt in the *TaCRR1* cDNA sequence) with *Nhe*I and *Mlu*I restriction sites was sub-cloned from the cDNA sequence of the CI12633 and inserted into the RNA γ chain of BSMV genomes in antisense orientation for gene silencing (Figure 6A). The Si-Fi software was used for the off-target prediction of the VIGS construct. Following Holzberg et al. [41] and Zhu et al. [6], the tripartite cDNA chains of BSMV virus genomes were separately linearized and transcribed into RNAs. Then, they were mixed and then inoculated onto the leaves of *R. cerealis*-resistant wheat cv. CI12633 or *B. sorokiniana*-resistant wheat cv. Yangmai 6 at the three leaf stage. Inoculated plants were grown at ~90% relative humidity for 7 days.

The RNA transcript of the BSMV *CP* gene was used as an index to examine if BSMV successfully infected the wheat plants using primers BSMV-CP-F/BSMV-CP-R (Table 1) at 10 days after transfection of BSMV. To detect if the transcriptional level of *TaCRR1* was down-regulated in silenced plants, the expression level of *TaCRR1* was analyzed by qRT-PCR using TaCRR1-QF/ TaCRR1-QR primers (Table 1). These CI12633 plants and Yangmai 6 plants were further inoculated with small toothpicks harboring the well-developed mycelia of *R. cerealis* or *B. Sorokiniana* at ~20 days after transfection of BSMV. Inoculated plants were grown at ~80% relative humidity. The disease symptoms of these plants were observed at 15 dpi, and ITs and sharp eyespot/common root rot disease indexes of these plants were scored at 35 dpi [6,27,28].

## Figures and Tables

**Table 1:** Primers and their sequences in this study.

Primer Name	Sequence (5′–3′)	Use
TaCRR1-F1	CGCACTCGTTATAAGCTGCC	PCR for *TaCRR1* DNA and cDNA amplification
TaCRR1-R1	ACAGCTTACATAACCCCCGC	
TaCRR1-F2	CACATGGCAAGCTCAATCGT	
TaCRR1-R2	GCATCTCAACACCAAAGGCA	
TaCRR1-QF	CTACCTATCCGACGCCAGAA	qRT-PCR for wheat *TaCRR1* transcript
TaCRR1-QR	CGTAGATGGTGACGAACGGC	
TaCRR1-γ-F	CTAGCTAGCAGGGTGCGCTACGAGATCTA	BSMV:*TaCRR1* vector construction
TaCRR1-γ-R	CTAGCTAGC TGCAGCTCAGATATCACGTCT	
BSMV-CP-F	TGACTGCTAAGGGTGGAGGA	RT-PCR for wheat BMSV coat protein
BSMV-CP-R	CGGTTGAACATCACGAAGAGT	
TaGAPDH-QF	TTAGACTTGCGAAGCCAGCA	qRT-PCR for wheat GAPDH transcript
TaGAPDH-QR	AAATGCCCTTGAGGTTTCCC	
Defensin-QF	ATGTCCGTGCCTTTTGCTA	qRT-PCR for wheat Defensin transcript
Defensin-QR	CCAAACTACCGAGTCCCCG	
Chitinase1-QF	ATGCTCTGGGACCGATACTT	qRT-PCR for wheat Chitinase1 transcript
Chitinase1-QR	AGCCTCACTTTGTTCTCGTTTG	
Chitinase3-QF	CCCACCCTAACCTGAGCATC	qRT-PCR for wheat *Chitinase3* transcript
Chitinase3-QR	ACTGGTTGATCATGGCGGAG	
Chitinase4-QF	GAAGTCCCCCATGGCGATC	qRT-PCR for wheat Chitinase4 transcript
Chitinase4-QR	GGTCCCGCAATAACCGTACT	
β-1,3-Glucanase-QF	ACGACATCACGGCGAGGT	qRT-PCR for wheat β-1,3-Glucanase transcript
β-1,3-Glucanase-QR	CACGGGGAAAGAGAGGATGA	
Pcold-TaCRR1-F	GGTACCCTCGAGGGATCCATGGCAAGCTCAATCGCT	Heterologously-expressed His-TF-TaCRR1 vector construction
Pcold-TaCRR1-R	AAGCTTGAATTCGGATCCTCAAGCGTGCACGACGAT	

## Supplementary Materials

Supplementary materials can be found at https://www.mdpi.com/1422-0067/21/16/5698/s1. Figure S1: Transcriptional patterns of *TaCRR1* in four wheat cultivars with different sharp eyespot-resistance degrees at 2 dpi with *R. cerealis* WK207. The transcriptional level of *TaCRR1* in the highly-susceptible wheat cultivar Wenmai 6 was set to 1. *TaGAPDH* was used as an internal control. Figure S2: Alignment of protein sequences of TaCRR1 with TaCRR1B and TaCRR1D. The software DANMAN was used to perform the sequence alignment.
