# Supplementary figures and images for: The Cysteine-Rich Repeat Protein TaCRR1 Participates in Defense against Both Rhizoctonia cerealis and Bipolaris sorokiniana in Wheat
Source: Int J Mol Sci. 2020 Aug 9;21(16):5698. doi: 10.3390/ijms21165698 (PMC7461100; doi:10.3390/ijms21165698)

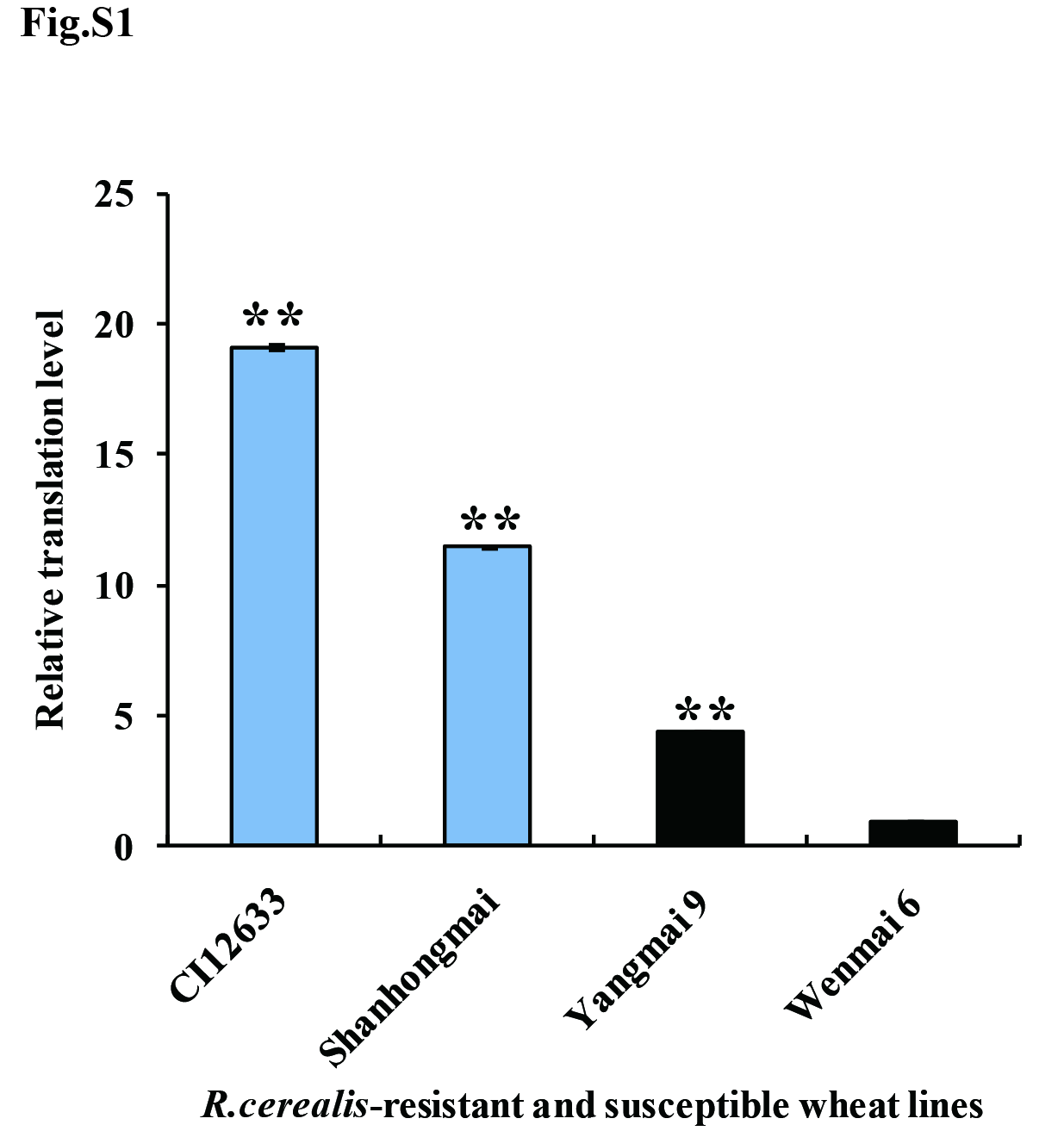

Supplement: Supplementary file 1 [file ijms-21-05698-s001.zip › Supplementary figure/Fig.S1.tif]

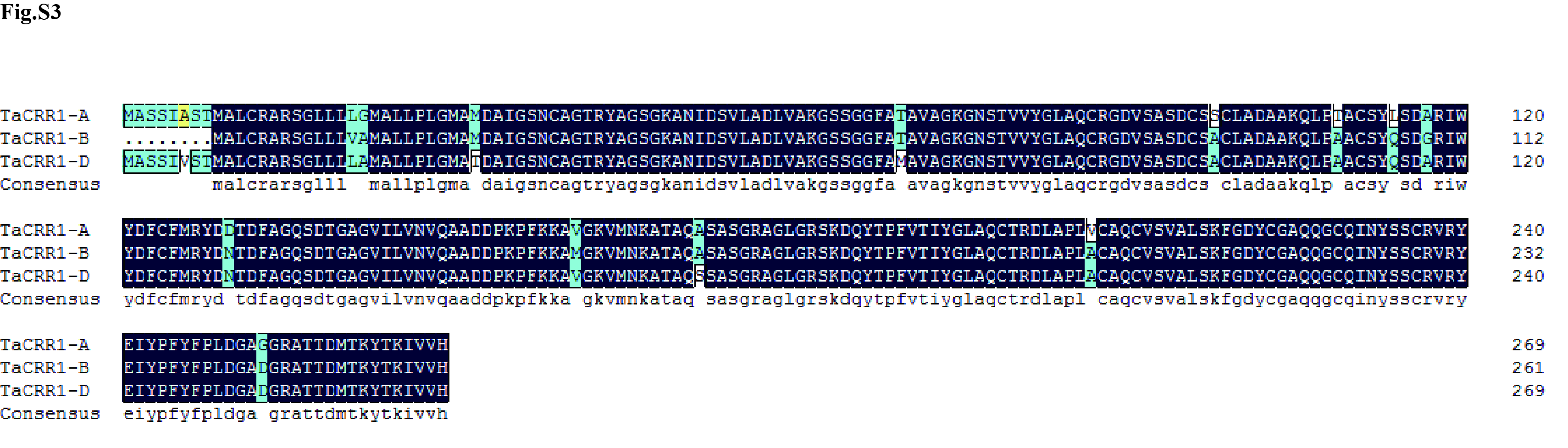

Supplement: Supplementary file 1 [file ijms-21-05698-s001.zip › Supplementary figure/Fig.S2.tif]
